# The association of crash response times and deaths at the crash scene: A cross‐sectional analysis using the 2019 National Emergency Medical Service Information System

**DOI:** 10.1111/jrh.12666

**Published:** 2022-04-22

**Authors:** Oluwaseun J. Adeyemi, Rajib Paul, Charles DiMaggio, Eric Delmelle, Ahmed Arif

**Affiliations:** ^1^ Department of Emergency Medicine New York University Grossman School of Medicine New York New York USA; ^2^ Department of Public Health Sciences University of North Carolina at Charlotte Charlotte North Carolina USA; ^3^ School of Data Science University of North Carolina at Charlotte Charlotte North Carolina USA; ^4^ Department of Surgery New York University Grossman School of Medicine New York New York USA; ^5^ Department of Population Health New York University Grossman School of Medicine New York New York USA; ^6^ Department of Geography and Earth Sciences University of North Carolina at Charlotte Charlotte North Carolina USA; ^7^ Department of Geographical and Historical Studies University of Eastern Finland Joensuu Finland

**Keywords:** crash response time, deaths at the crash scene, EMS travel time, rurality/urbanicity, rush hour

## Abstract

**Background:**

Deaths at the crash scene (DAS) are crash deaths that occur within minutes after a crash. Rapid crash responses may reduce the occurrence of DAS.

**Objectives:**

This study aims to assess the association of crash response time and DAS during the rush and nonrush hour periods by rurality/urbanicity.

**Method:**

This single‐year cross‐sectional study used the 2019 National Emergency Medical Services (EMS) Information System. The outcome variable was DAS. The predictor variables were crash response measures: EMS Chute Initiation Time (ECIT) and EMS Travel Time (ETT). Age, gender, substance use, region of the body injured, and the revised trauma score were used as potential confounders. Logistic regression was used to assess the unadjusted and adjusted odds of DAS.

**Results:**

A total of 654,675 persons were involved in EMS‐activated road crash events, with 49.6% of the population exposed to crash events during the rush hour period. A total of 2,051 persons died at the crash scene. Compared to the baseline of less than 1 minute, ECIT ranging from 1 to 5 minutes was significantly associated with 58% (95% CI: 1.45‐1.73) increased odds of DAS. Also, when compared to the baseline of less than 9 minutes, ETT ranging between 9 and 18 minutes was associated with 34% (95% CI: 1.22‐1.47) increased odds of DAS. These patterns were consistent during the rush and nonrush hour periods and across rural and urban regions.

**Conclusion:**

Reducing crash response times may reduce the occurrence of DAS.

## INTRODUCTION

Road crashes remain a preventable cause of death in the United States. As of 2019, 36,096 crash fatalities were recorded in the United States, representing 1.1 fatalities per 100,000 vehicle miles traveled.^1^ Approximately 1 person dies in a crash every 14 minutes in the United States.^2^ The United States experienced a decline in fatal deaths between 2016 and 2019.^1^, ^3^ However, fatal crash estimates in 2020 and 2021 were the highest the United States had experienced in the last 2 decades.^4^, ^5^, ^6^

The rush hour period represents the period of peak road activities. The period varies widely across rural and urban regions, with the peak densities occurring between 6‐9 am and 3‐7 pm.^7^, ^8^, ^9^ About a quarter of fatal crash injuries occur during the rush hour period.^10^, ^11^ Also, there are rural‐urban differences in crash occurrences and characteristics, with rural areas having longer crash response times and increased crash fatality rates.^12^, ^13^

Deaths at the crash scene (DAS) represent a unique subset of salvageable and unsalvageable crash deaths that would have occurred within minutes after the crash, probably due to damage to vital structures.^14^ Although DAS is an infrequently reported crash characteristic, cases of death on arrival at the emergency department have been used in previous studies as a proxy in determining cases that either died at the crash scene or in transit.^15^, ^16^, ^17^

Central to preventing fatal crash events is a rapid crash response.^13^ Acute blood loss, one of the major clinical presentations of crash injury victims, is a time‐dependent diagnosis that requires interventional care within minutes of its occurrence.^18^ The Emergency Medical Services (EMS) crash response can be conceptualized to occur in 4 nonoverlapping temporal phases: the period from crash occurrence to EMS notification, from EMS notification to chute initiation, from EMS chute initiation to crash scene arrival, and from crash scene arrival to hospital arrival.^19^, ^20^ Delay at any of these phases may potentially increase the odds of unfavorable health outcomes.

While acknowledging that some crash injuries may be unsalvageable,^15^ there is compelling evidence that the reduction in crash response time is associated with improved crash injury survival.^13^, ^21^, ^22^ It is unknown how DAS varies in rural and urban areas during rush and nonrush hours. To date, no study in the public domain has assessed the association between crash response times and DAS. This study aims to estimate the occurrence of cases classified as DAS and how this occurrence varies during the rush and nonrush hour periods and by rurality/urbanicity. Additionally, this study will assess the association of measures of EMS crash response time and DAS at all times of the day and during the rush and nonrush hour periods by rurality/urbanicity.

## METHODS

### Study design

This population‐based single‐year study used the 2019 National Emergency Medical Services Information System (NEMSIS), a census of all the EMS activations across the continental United States, excluding Idaho, Missouri, Massachusetts, and Ohio.^23^ Cases in the NEMSIS represent trauma and nontrauma emergency cases, pooled from all regional EMS agencies.^23^, ^24^

### Inclusion and exclusion criteria

**FIGURE 1:**
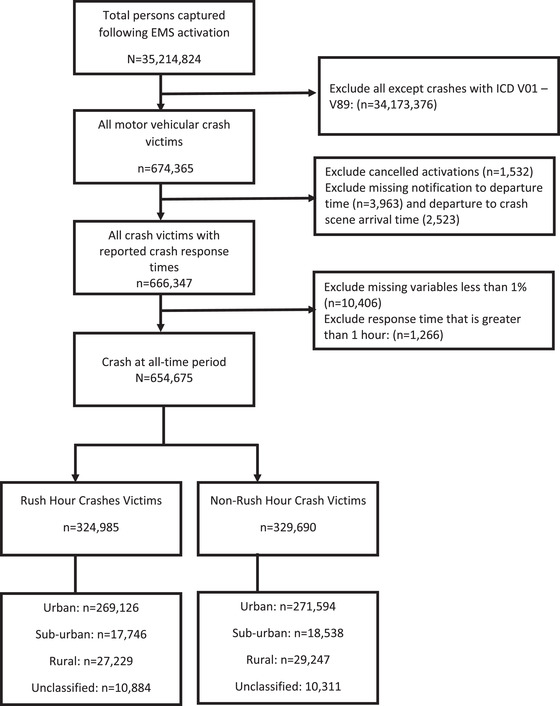
Data selection steps

A total of 35,214,824 persons were involved in all EMS activations. All road crashes were selected. Crashes were identified using the International Classification of Disease (ICD) code, version 10 (Figure 1). Specifically, cases identified as ICD V01‐V89 were selected. These cases represented crashes involving pedestrians (V01‐V09), pedal cyclists (V10‐V19), motorcycle (V20‐V29), 3‐wheeled motor (V30‐V39), cars (V40‐V49), trucks (V50‐V59), heavy transport vehicle (V60‐V69), bus (V70‐V79), and other land transport (V80‐V89) (n = 674,365). Cases whose crash outcome was classified as “canceled” were excluded (n = 1,532). Also, we excluded cases whose crash response times were over 1 hour (n = 1,266). These cases were excluded because they represent outliers cases that occurred during unique situations. For example, a crash event in Alabama that occurred on March 3 had a response time of 6 hours but the event occurred during a tornado that involved 41 locations.^25^ The NEMSIS did not provide context for all the unexpectedly prolonged response times so we excluded cases whose response time exceeded 1 hour. Additionally, we excluded cases with missing crash notification to EMS base station departure time (n = 3,963) and missing EMS base station departure time to crash scene arrival time (2,523). Also, we excluded cases when the predictor and control variables have missingness less than 1% (n = 10,466). The final sample included 654,675 persons involved in all forms of road crashes.

### Rush and nonrush hour period

Rush hour period was defined as crash injuries occurring between 6‐9 am and 3‐7 pm.^9^ The nonrush hour period represents the intervening period of the day and night that excludes the times defined as the rush hour period. We classified persons involved in the crashes into the rush and nonrush hour period based on the hour the EMS was notified.

### Rurality/urbanicity categorization

The geographical location of the persons involved in the crash was reported as a 4‐point categorical variable: urban, suburban, rural, and wilderness. We recoded this variable into 3 categories, combining rural and wilderness in a single category (hereafter referred to as rural). We performed a separate analysis for cases that occurred in rural, suburban, and urban areas.

### Outcome variable: death at the crash scene

The main outcome variable was DAS, defined as cases classified as dead at the incident by EMS personnel. The original variable used to measure DAS was “e‐disposition.12.” eDisposition.12 describes the incident patient disposition, and it includes cases that were not treated and discharged, treated and discharged at the scene, refused care, treated and transported either by the EMS or other forms of transport, were assisted by the EMS and non‐EMS personnel, and those that died at the scene with or without resuscitation. Consistent with our definition of DAS, deaths reported in eDisposition.12 did not include deaths while in transit to the hospital. eDisposition.12 was recoded into 2 categories: died at the crash scene and otherwise.

### Predictor variables: crash response times

Two crash response measures were used as predictor variables. The first predictor was EMS Chute Initiation Time (ECIT), defined as the duration from EMS notification to the EMS team departure from its base (“EMS Chute Time Mins”). The ECIT had been computed in the NEMSIS dataset as a continuous variable, measured in minutes. This variable was defined as a 3‐point categorical variable: less than 1 minute, 1‐5 minutes, and more than 5 minutes. The second predictor was the EMS Travel Time (ETT) defined as the duration from the EMS team's departure from its base (“EMS Scene Response Time Min”) to the time the team arrived at the crash scene (eTimes.06). The ETT had been computed in the NEMSIS dataset as a continuous variable, measured in minutes. This variable was defined as a 4‐point categorical variable: less than 9 minutes, 9‐17.9 minutes, 18‐26.9 minutes, and 27 minutes or higher. These categorizations were informed by the benchmark set by the National Fire Protection Agency and the Fire and EMS Department, which requires the “turnout” time to be a minute or less and EMS travel time to be less than 9 minutes.^26^, ^27^ Earlier studies have used other crash response measures, such as crash occurrence to EMS notification time and crash scene arrival to hospital arrival time, as predictors of fatal crash injury.^12^, ^13^ We did not use crash occurrence to EMS notification time since the NEMSIS does not report the time the crash occurred.^20^ Since we were interested in the events that precede DAS, crash scene arrival to hospital arrival time was not used.

### Confounding

Age, gender, substance use, region of the body injured, and the revised trauma score (RTS) were used as potential confounders. The region of the body injured was recoded into 4 categories: (1) head and neck, (2) chest and back, (3) abdomen, pelvis, and limbs, and (4) multiple body injuries. RTS, a measure of injury severity, was computed using the Glasgow Coma Scale score (GCS), respiratory rate (RR), and systolic blood pressure (SBP). These 3 variables were recategorized into 5 categories ranging from 0 to 4 as defined in the original documentation.^28^ The final RTS was calculated as the sum of (0.9368^*^GCS category) + (0.7326^*^SBP category) + (0.2908^*^RR category). Missingness in the RTS was addressed by using the EMS final patient disposition as a proxy. The EMS final patient disposition, a variable that captures the clinical outcome after EMS intervention, was measured as a 4‐point categorical variable: critical, emergent, low priority, and dead. To address the missing values in RTS, missing values in the GCS, RR, and SBP categories identified as a low priority were given a score of 4 since these patients would have stable vital signs, otherwise would not be categorized as low priority. Also, missing values in GCS, RR, and SBP categories that were categorized as critical, emergent, or dead were assigned values of 3, 2, and 1, respectively.

### Analysis

Frequency distribution of the sociodemographic, crash, injury, and crash response characteristics were computed across the total population and the DAS and the alive/non‐DAS death categories. Also, the frequency distribution of the study population and the population categorized as DAS was assessed across the rush and nonrush hour periods. The prevalence of DAS was computed across all times of the day and during the rush and nonrush hour periods and by rurality/urbanicity. Univariate and multivariate logistic regression analysis was performed to assess the relationship between the crash response time and DAS. Separate analyses were conducted by rurality/urbanicity and rush and nonrush hour periods. Data were analyzed using STATA version 16.^29^

## RESULTS

Of the 654,675 persons included in the study, a total of 2,051 persons, representing 0.3% of all the persons involved in crashes, were classified as DAS, while the remaining 652,624 persons (99.7%) were either alive or had non‐DAS deaths (Table 1). The majority of the population were aged 36‐55 years (27.8%) with an approximately equal male‐female distribution. Multiple injuries over the general body (24.1%) followed by injury to the abdomen, pelvis, and limbs (19.5%) were the commonly injured body parts. Substance use‐associated crash injury represented about 7.2% of the cases, and most of the crash cases occurred in the urban areas (82.6%). The median (Q1, Q3) RTS was 7.84 (7.25, 7.84). About 64% of crashes had ECIT of less than 1 minute and about two‐thirds of the crashes had an ETT of less than 9 minutes. There were statistically significant associations between DAS and age, sex, injured region of the body, substance use, the geographical location of the crash, RTS, ECIT, and ETT (*P* < .001).

**TABLE 1:** Frequency distribution and summary statistics of the EMS crash response times, sociodemographic, clinical, and location‐based characteristics assessed across mortality status

	All period (N = 654,675)	Death‐at‐the‐scene (n = 2,051 [0.3%])	Alive/other deaths (n = 652,624 [99.7%])	
Variables	Frequency (%)	Frequency (%)	Frequency (%)	*P*‐value*
Age categories				
< 16 years	54,565 (8.3)	59 (2.9)	54,506 (8.4)	<.001
16‐25 years	144,087 (22.0)	390 (19.0)	143,697 (22.0)	
26‐35 years	126,160 (19.3)	457 (22.3)	125,703 (19.3)	
36‐55 years	182,286 (27.8)	624 (30.4)	181,662 (27.8)	
56‐75 years	121,135 (18.5)	419 (20.4)	120,716 (18.5)	
>75 years	26,442 (4.0)	102 (5.0)	26,340 (4.0)	
Sex				
Male	323,450 (49.4)	1,479 (72.1)	321,971 (49.3)	<.001
Female	331,225 (50.6)	572 (27.9)	330,653 (50.7)	
Injured region				
Head and neck	110,327 (16.9)	167 (8.1)	110,160 (16.9)	<.001
Chest and back	88,696 (13.6)	57 (2.8)	88,639 (13.6)	
Abdomen, pelvis, and limbs	127,857 (19.5)	8 (0.4)	127,849 (19.6)	
General body	158,057 (24.1)	1,080 (52.7)	156,977 (24.0)	
Unknown	169,738 (25.9)	739 (36.0)	168,999 (25.9)	
Substance use				
Yes	47,420 (7.2)	111 (5.4)	47,309 (7.3)	<.001
No	432,069 (66.0)	1,028 (50.1)	431,041 (66.0)	
Unknown	175,186 (26.8)	912 (44.5)	174,274 (26.7)	
Geographical location				
Rural/wilderness	56,476 (8.6)	354 (17.3)	56,122 (8.6)	<.001
Suburban	36,284 (5.6)	196 (9.6)	36,088 (5.5)	
Urban	540,720 (82.6)	1,437 (70.1)	539,283 (82.6)	
Unknown	21,195 (3.2)	64 (3.0)	21,131 (3.3)	
Revised trauma score				
Mean (SD)*	7.61 (5.79)	3.39 (1.64)	7.63 (0.58)	<.001
Median (Q1, Q3) **	7.84 (7.25, 7.84)	3.80 (2.93, 4.74)	7.84 (7.55, 7.84)	<.001
EMS chute initiation time (ECIT)				
Less than 1 minute	420,958 (64.3)	1,116 (54.4)	419,842 (64.3)	<.001
1‐5 minutes	207,894 (31.8)	871 (42.5)	207,023 (31.7)	
More than 5 minutes	25,823 (3.9)	64 (3.1)	25,759 (4.0)	
EMS travel time (ETT)				
Less than 9.0 minutes	429,614 (65.6)	1,193 (58.2)	428,421 (65.7)	<.001
9.0‐17.9 minutes	176,201 (26.9)	655 (31.9)	175,546 (26.9)	
18.0‐26.9 minutes	33,704 (5.2)	140 (6.8)	33,564 (5.1)	
27 minutes or higher	15,156 (2.3)	63 (3.1)	15,093 (2.3)	

* Independent sample *T*‐test performed.

** Mann‐Whitney U test performed.

The number of persons who sustained crash injuries during the rush (n = 324,985 [49.6%]) and nonrush hour (n = 329,690 [50.4%]) periods was almost equal (Table 2). However, there were statistically significant differences in the sociodemographic and crash characteristics during the rush and nonrush hour periods. A larger proportion of crashes during the rush hour period involved individuals aged 35‐55 years (rush hour [RH]: 28.4% vs nonrush hour [NRH]: 27.2%). More females were involved in rush hour‐related (52.6%) crashes than in nonrush hour‐related crashes (48.6%; *P* < .001). Injuries to the head and neck (RH: 17.1% vs NRH: 16.6%) were significantly higher during the rush hour period as compared to the nonrush hour period (*P* < .001). The proportion of cases associated with substance use in the rush hour period (4.7%) was less than half the proportion of cases occurring in the nonrush hour period (9.8%; *P* < .001). Further, the proportion of urban‐related cases was marginally higher during the rush hour period than the nonrush hour period (RH: 82.8% vs NRH: 82.4%; *P* < .001). The proportion of crashes with ECIT less than a minute was higher during the rush hour period than in the nonrush hour period (RH: 65.8% vs NRH: 62.9%; *P* < .001). Similarly, the proportion of crashes with an ETT of less than 9 minutes was marginally higher during the rush hour period than in the nonrush hour period (RH: 65.8% vs NRH: 65.4%; *P* < .001). The proportion of cases classified as DAS was fewer during the rush hour period compared to the nonrush hour period (RH: 0.2% vs NRH: 0.4%; *P* < .001). When we restricted the data to cases classified as DAS, the statistically significant differences in the rush and nonrush hour periods and age, sex, substance use, and the geographical location of the crash persisted.

**TABLE 2:** Frequency distribution and summary statistics of the EMS crash response times, sociodemographic, clinical, and location‐based characteristics at all times and during the rush and nonrush hour periods and among crash victims who died at the crash scene

	Rush hour (n = 324,985 [49.6%])	Nonrush hour (n = 329,690 [50.4%])		Death‐at‐the‐scene: rush hours (n = 762 [37.1%])	Death‐at‐the‐scene: nonrush hours (n = 1,289 [62.9%])	
Variables	Frequency (%)	Frequency (%)	*P*‐value	Frequency (%)	Frequency (%)	*P*‐value
Age categories						
< 16 years	31,470 (9.7)	23,095 (7.0)	<.001	33 (4.3)	26 (2.0)	.002
16‐25 years	67,233 (20.7)	76,854 (23.3)		139 (18.3)	251 (19.5)	
26‐35 years	60,997 (18.8)	65,163 (19.8)		151 (19.8)	306 (23.7)	
36‐55 years	92,471 (28.4)	89,815 (27.2)		225 (29.5)	399 (31.0)	
56‐75 years	60,460 (18.6)	60,675 (18.4)		165 (21.7)	254 (19.7)	
>75 years	12,354 (3.8)	14,088 (4.3)		49 (6.4)	53 (4.1)	
Sex						
Male	154,051 (47.4)	169,399 (51.4)	<.001	522 (68.5)	957 (74.2)	.005
Female	170,934 (52.6)	160,291 (48.6)		240 (31.5)	332 (25.8)	
Injured region						
Head and neck	55,573 (17.1)	54,754 (16.6)	<.001	54 (7.1)	113 (8.8)	.318***
Chest and back	45,022 (13.9)	43,674 (13.2)		24 (3.1)	33 (2.6)	
Abdomen, pelvis, and limbs	63,548 (19.5)	64,309 (19.5)		3 (0.4)	5 (0.4)	
General body	76,134 (23.4)	81,923 (24.9)		389 (51.1)	691 (53.6)	
Unknown	84,708 (26.1)	85,030 (25.8)		292 (38.3)	447 (34.7)	
Substance use						
Yes	15,142 (4.7)	32,278 (9.8)	<.001	31 (4.1)	80 (6.2)	.035
No	222,847 (68.6)	209,222 (63.5)		404 (53.0)	624 (48.4)	
Unknown	89,996 (26.7)	88,190 (26.7)		327 (42.9)	585 (45.4)	
Geographical location						
Rural/wilderness	27,229 (8.4)	29,247 (8.9)	<.001	158 (20.7)	196 (15.2)	.016
Suburban	17,746 (5.5)	18,538 (5.6)		69 (9.1)	127 (9.8)	
Urban	269,126 (82.8)	271,594 (82.4)		513 (67.3)	924 (71.7)	
Unknown	10,884 (3.3)	10,311 (3.1)		22 (2.9)	42 (3.3)	
Revised trauma score						
Mean (SD)*	7.63 (0.59)	7.59 (0.67)	<.001	3.43 (1.68)	3.37 (1.62)	.373
Median (Q1, Q3) **	7.84 (7.55, 7.84)	7.84 (7.55, 7.84)	<.001	3.80 (2.93, 4.74)	3.80 (2.93, 4.74)	.270
EMS chute initiation time (ECIT)						
Less than 1 minute	213,728 (65.8)	207,230 (62.9)	<.001	435 (57.1)	681 (52.8)	.115
1‐5 minutes	99,496 (30.6)	108,398 (32.9)		308 (40.4)	563 (43.7)	
More than 5 minutes	11,761 (3.6)	14,062 (4.3)		19 (2.5)	45 (3.5)	
EMS travel time (ETT)						
Less than 9.0 minutes	213,852 (65.8)	215,762 (65.4)	<.001	423 (55.5)	770 (59.7)	.301
9.0‐17.9 minutes	87,856 (27.0)	88,345 (26.8)		258 (33.9)	397 (30.8)	
18.0‐26.9 minutes	16,411 (5.1)	17,293 (5.3)		57 (7.5)	83 (6.4)	
27 minutes or higher	6,866 (2.1)	8,290 (2.5)		24 (3.1)	39 (3.0)	
Mortality status						
Death‐at‐the‐scene	762 (0.2)	1,289 (0.4)	<.001			
Alive/other death types	324,223 (99.8)	328,401 (99.6)				

* Independent sample *T*‐test performed.

** Mann‐Whitney U test performed.

*** Fisher's exact performed.

**FIGURE 2:**
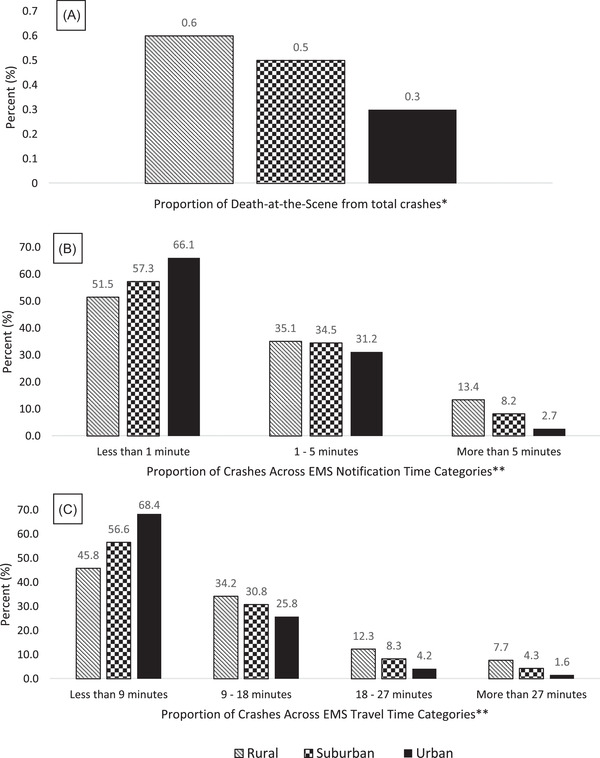
Geographical distribution of (A) deaths at the crash scene, (B) EMS notification time, and (C) EMS travel time. ^*^The corresponding counts (n) of deaths at the crash scene are: rural/wilderness: 339, suburban: 181, and urban: 1,623. The corresponding counts (%) of those alive/not dead at the crash scene are as follows: rural/wilderness: 56,122 (99.4%), suburban: 36,044 (99.5%), and urban: 539,283 (99.7%). There were statistically significant differences in the deaths at the scene across geographical location (*P* < .001). ^**^There were statistically significant differences across the geographical locations by EMS notification time (*P* < .001) and by EMS travel time (*P* < .001)

As one migrates from urban to rural areas, the proportion of DAS significantly increases. About 0.3% of persons involved in crashes in urban environments were classified as DAS (Figure 2A). The proportion of DAS increases to 0.5% and 0.6% in suburban and rural areas. Also, the proportion of persons involved in crashes with delayed EMS response times significantly increases as one migrates from urban to rural areas. About 2.7% of all persons involved in crashes in the urban areas experienced an ECIT delay of over 5 minutes, while the proportion of persons involved in crashes with delayed ECIT occurring over 5 minutes was 8.2% and 13.4% in suburban and rural areas, respectively (Figure 2B). Similarly, about 1.6% of persons involved in crashes experienced a delay in ETT of over 27 minutes in urban areas, while the proportion of persons involved in crashes who experienced a delay in ETT of over 27 minutes was 4.3% and 7.3% in suburban and rural areas, respectively (Figure 2C).

Age, gender, injured body region, geographical location of the injury, and RTS were significantly associated with DAS at all times of the day and the rush and nonrush hour periods (Table 3). Across all times of the day and during the rush hour period, the unadjusted odds of DAS increased with increasing age. During the rush and nonrush hour periods, crash victims older than 75 years were 3.8 times (OR: 3.79; 95% CI: 2.44‐5.90) and 3.4 times (OR: 3.35; 95% CI: 2.09‐5.36) more likely to be classified as DAS, respectively. Males were 2.7 and 2.4 times more likely to be classified as DAS compared to females during the all‐time (OR: 2.66; 95% CI: 2.41‐2.92) and rush hour periods (OR: 2.42; 95% CI: 2.08‐2.82). Persons classified as DAS were significantly less likely to sustain head and neck injuries as compared to multiple body injuries at all‐time of the day (OR: 0.22; 95% CI: 0.19‐0.26) and during the rush (OR: 0.19; 95% CI: 0.14‐0.25) and nonrush hour periods (OR: 0.24; 95% CI: 0.20‐0.30). At all times of the day, persons exposed to crashes in rural (OR: 2.37; 95% CI: 2.11‐2.66) and suburban (OR: 2.04; 95% CI: 1.75‐2.37) areas were 2 times more likely to be classified as DAS. During the rush hour period, the unadjusted odds of DAS were heightened with persons exposed to crashes in rural and suburban areas being 3 times (OR: 3.06; 95% CI: 2.56‐3.65) and 2 times (OR: 2.04; 95% CI: 1.59‐2.63) more likely to be classified as DAS, respectively. A unit increase in RTS was associated with reduced odds of DAS (OR: 0.27; 95% CI: 0.27‐0.28) at all times of the day and these odds were similar during the rush and nonrush hour periods.

**TABLE 3:** Summary of the unadjusted odds of dead‐at‐scene (DAS) associated with EMS response times, sociodemographic, clinical, and location‐based characteristics, measured across all periods and the rush and nonrush hour periods

	All period (n = 654,675)	Rush hour (n = 324,985)	Nonrush hour (n = 329,690)
Variables	Odds ratio (95% CI)	Odds ratio (95% CI)	Odds ratio (95% CI)
Age			
16‐25 years	**2.51 (1.91**‐**3.30)**	**1.97 (1.35**‐**2.89)**	**2.91 (1.94**‐**4.35)**
26‐35 years	**3.36 (2.56**‐**4.41)**	**2.36 (1.62**‐**3.45)**	**4.19 (2.80**‐**6.25)**
36‐55 years	**3.17 (2.43**‐**4.15)**	**2.32 (1.61**‐**3.35)**	**3.96 (2.66**‐**5.89)**
56‐75 years	**3.21 (2.44**‐**4.21)**	**2.61 (1.79**‐**3.79)**	**3.72 (2.49**‐**5.59)**
>75 years	**3.58 (2.60**‐**4.93)**	**3.79 (2.44**‐**5.90)**	**3.35 (2.09**‐**5.36)**
< 16 years	Ref	Ref	Ref
Gender			
Male	**2.66 (2.41**‐**2.92)**	**2.42 (2.08**‐**2.82)**	**2.74 (2.42**‐**3.10)**
Female	Ref	Ref	Ref
Injured region			
Head and neck	**0.22 (0.19**‐**0.26)**	**0.19 (0.14**‐**0.25)**	**0.24 (0.20**‐**0.30)**
Chest and back	**0.09 (0.07**‐**0.12)**	**0.10 (0.07**‐**0.16)**	**0.09 (0.06**‐**0.13)**
Abdomen, pelvis, and limbs	**0.01 (0.00**‐**0.02)**	**0.01 (0.00**‐**0.03)**	**0.01 (0.00**‐**0.02)**
Multiple body injury	Ref	Ref	Ref
Substance use			
Yes	0.98 (0.81‐1.20)	1.13 (0.78‐1.63)	0.83 (0.66‐1.04)
No	Ref	Ref	Ref
Geographical location			
Rural/wilderness	**2.37 (2.11**‐**2.66)**	**3.06 (2.56**‐**3.65)**	**1.98 (1.69**‐**2.31)**
Suburban	**2.04 (1.75**‐**2.37)**	**2.04 (1.59**‐**2.63)**	**2.02 (1.68**‐**2.43)**
Urban	Ref	Ref	Ref
Revised trauma score	**0.27 (0.27**‐**0.28)**	**0.28 (0.27**‐**0.29)**	**0.27 (0.26**‐**0.28)**
EMS chute initiation time (ECIT)			
More than 5 minutes	**1.58 (1.45**‐**1.73)**	**1.52 (1.32**‐**1.76)**	**1.58 (1.42**‐**1.77)**
1‐5 minutes	0.93 (0.73‐1.20)	0.79 (0.50‐1.26)	0.97 (0.72‐1.32)
Less than 1 minute	Ref	Ref	Ref
EMS travel time (ETT)			
9.0‐17.9 minutes	**1.34 (1.22**‐**1.47)**	**1.49 (1.27**‐**1.74)**	**1.26 (1.12**‐**1.42)**
18.0‐26.9 minutes	**1.50 (1.26**‐**1.79)**	**1.76 (1.33**‐**2.32)**	**1.35 (1.07**‐**1.69)**
27 minutes or higher	**1.50 (1.16**‐**1.93)**	**1.77 (1.17**‐**2.67)**	1.32 (0.96‐1.82)
Less than 9.0 minutes	Ref	Ref	Ref

In the unadjusted model, delay in ECIT by more than 5 minutes was associated with increased odds of DAS (Table 3). When compared to ECIT that occurred in less than 1 minute, ECIT ranging from 1 to 5 minutes was significantly associated with 58% (95% CI: 1.45‐1.73) and 52% (95% CI: 1.32‐1.76) increased odds of DAS at all times of the day and during the rush hour period. After adjusting for potential confounders, ECIT ranging from 1 to 5 minutes was significantly associated with 45% (95% CI: 1.30‐1.63) and 50% (95% CI: 1.25‐1.81) increased odds of DAS at all times of the day and during the rush hour period (Table 4). However, ECIT occurring after 5 minutes was significantly associated with reduced odds of DAS at all times of the day and during the rush and nonrush hour periods.

**TABLE 4:** Summary of the adjusted logistic regression models across all time and during the rush hour period, estimating the odds of dead‐at‐scene (DAS) across different EMS response times

	All period	Rush hour	Nonrush hour
Variables	Adjusted odds ratio (95% CI)	Adjusted odds ratio (95% CI)	Adjusted odds ratio (95% CI)
EMS chute initiation time (ECIT)			
1‐5 minutes	**1.45 (1.30**‐**1.63)**	**1.50 (1.25**‐**1.81)**	**1.40 (1.21**‐**1.62)**
More than 5 minutes	**0.39 (0.29**‐**0.53)**	**0.33 (0.19**‐**0.55)**	**0.43 (0.30**‐**0.61)**
Less than 1 minute	Ref	Ref	Ref
EMS travel time (ETT)			
9.0‐17.9 minutes	**1.51 (1.33**‐**1.70)**	**1.75 (1.43**‐**2.12)**	**1.38 (1.18**‐**1.61)**
18.0‐26.9 minutes	**1.34 (1.08**‐**1.67)**	**1.60 (1.14**‐**2.26)**	1.21 (0.92‐1.60)
27 minutes or higher	0.84 (0.62‐1.14)	1.27 (0.80‐2.02)	0.64 (0.42‐0.96)
Less than 9.0 minutes	Ref	Ref	Ref

*Note*: Models adjusted for age, gender, injured parts, substance use, location of crash (rural/urban/suburban), and revised trauma score.

As the ETT increased, the unadjusted odds of DAS increased at all times of the day and during the rush and nonrush hour periods. When compared to ETT of less than 9 minutes, ETT ranging between 9 and 18 minutes was associated with 34% (95% CI: 1.22‐1.47), 49% (95% CI: 1.27‐1.74), and 26% (95% CI: 1.12‐1.42) increased odds of DAS at all times of the day and during the rush and nonrush hour periods, respectively. After adjusting for potential confounders, ETT ranging between 9 and 18 minutes was associated with 51% (95% CI: 1.33‐1.70), 75% (95% CI: 1.43‐2.12), and 38% (95% CI: 1.18‐1.61) increased odds of DAS at all times of the day and during the rush and nonrush hour periods, respectively.

**FIGURE 3:**
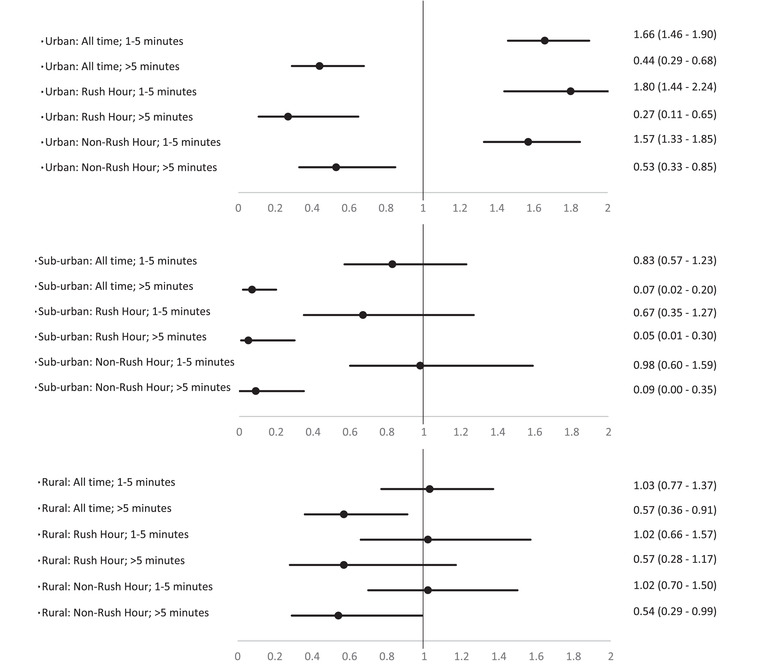
Adjusted odds ratio of dead‐at‐scene (DAS) from increasing EMS notification time measured across urban, suburban, and rural areas at all times, the rush hour and the nonrush hour periods. All models adjusted for age, gender, injured parts, substance use, and revised trauma score

In urban areas, the adjusted odds of DAS were significantly elevated when ECIT occurred between 1 and 5 minutes as compared to ECIT of less than 1 minute at all times of the day (AOR: 1.66; 95% CI: 1.46‐1.90) and during the rush (AOR: 1.80; 95% CI: 1.44‐2.24) and nonrush hour periods (AOR: 1.57; 95% CI: 1.33‐1.85) (Figure 3). However, when the ECIT occurred after 5 minutes, the adjusted odds of DAS were significantly reduced. In suburban areas, ECIT that occurred after 5 minutes were associated with reduced adjusted odds of DAS at all times of the day (AOR: 0.07; 95% CI: 0.02‐0.20) and during the rush hour (AOR: 0.05; 95% CI: 0.01‐0.30) and nonrush hour (AOR: 0.09; 95% CI: 0.00‐0.35) periods. In the rural areas, ECIT that occurred after 5 minutes were associated with reduced adjusted odds of DAS at all times of the day (AOR: 0.57; 95% CI: 0.36‐0.91) and during the nonrush hour period (AOR: 0.54; 95% CI: 0.29‐0.99).

**FIGURE 4:**
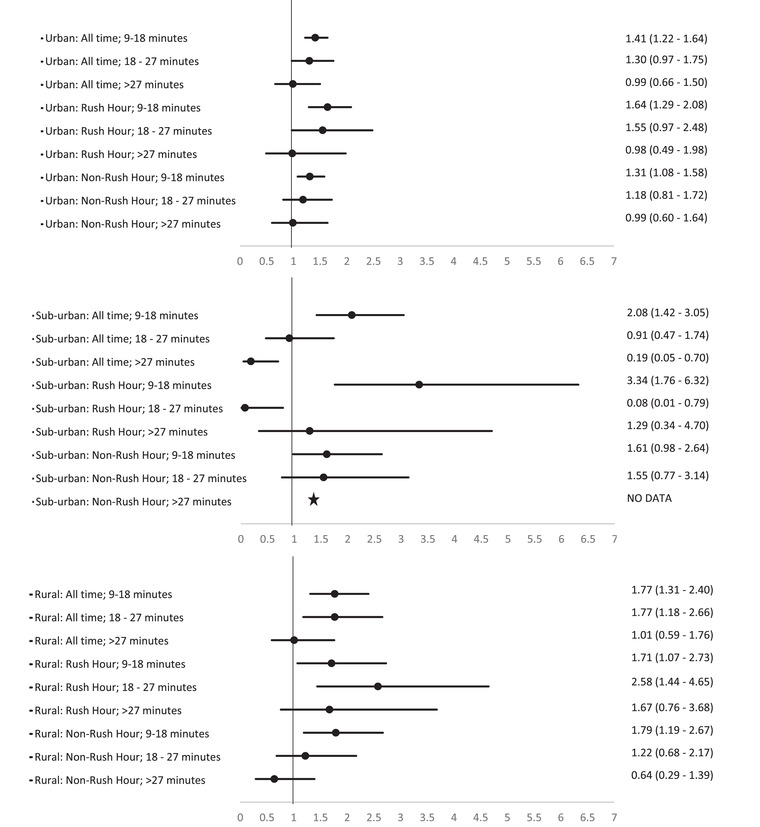
Adjusted odds ratio of dead‐at‐the‐scene from increasing EMS travel time measured across urban, suburban, and rural areas at all times, the rush hour and the nonrush hour periods. All models adjusted for age, gender, injured parts, substance use, and revised trauma score

In urban areas, the adjusted odds of DAS were significantly elevated when the duration of the ETT ranged between 9 and 18 minutes as compared to less than 9 minutes (Figure 4). This pattern of relationship was consistent at all times of the day (AOR: 1.41; 95% CI: 1.22‐1.64) and during the rush (AOR: 1.64; 95% CI: 1.29‐2.08) and nonrush hour (AOR: 1.31; 95% CI: 1.08‐1.58) periods in the urban areas. Similarly, in suburban areas, the adjusted odds of DAS were significantly elevated when the duration of the ETT ranged between 9 and 18 minutes as compared to less than 9 minutes at all times of the day (AOR: 2.08; 95% CI: 1.42‐3.05) and during the rush hour period (AOR: 3.34; 95% CI: 1.76‐6.32). Further, in rural areas, the adjusted odds of DAS were significantly elevated when the duration of the ETT ranged between 9 and 18 minutes as compared to less than 9 minutes at all times of the day (AOR: 1.77; 95% CI: 1.31‐2.40) during the rush hour (AOR: 1.71; 95% CI: 1.07‐2.73) and nonrush hour periods (AOR: 1.79; 95% CI: 1.19‐2.67).

## DISCUSSION

About half of the persons involved in crashes sustained crash injuries during the rush hour period and less than 10% of the crash population sustained crash injuries in the rural areas. Despite the low proportion of rush and nonrush hour crashes in the rural areas, the proportion of cases of DAS is highest in rural areas, and this proportion decreases stepwise from suburban to urban areas. A similar pattern was observed with crash response time, with the proportion of persons who encountered prolonged ECIT and ETT highest in rural areas and decreases stepwise from suburban to urban. Compared to ECIT less than 1 minute, ECIT exceeding the 1 minute but not more than 5 minutes was significantly associated with DAS, at all times of the day and during the rush and nonrush hour periods. However, ECIT exceeding 5 minutes was associated with reduced odds of DAS and the pattern holds at all times of the day and during the rush and nonrush hour periods. Compared to ETT less than 9 minutes, ETT between 9 and 18 minutes was associated with increased odds of DAS, and further multiples of this range were associated with a gradual attenuation of the odds of DAS. This pattern of association holds at all times of the day and during the rush and nonrush hour periods.

This study reports that as of 2019, approximately 2,000 persons were certified dead at the crash scene. This represents about 5% of all crash‐related deaths, using the 2019 National Highway Traffic Safety estimates.^1^, ^30^ Although the proportion of DAS was lower during the rush hour period compared to the nonrush hour period, the odds of DAS were heightened during the rush hour period. A recent meta‐analysis reported that the rush hour period is associated with increased odds of fatal crash injuries.^9^ The possibility exists that certain rush hour‐related risky driving behaviors may be responsible for the heightened odds of DAS. While we controlled for substance use, we were unable to control for other risky driving behaviors like speeding, seatbelt use, and phone‐related distracted driving.

In this study, ECIT was defined as the duration from a 911 call to the time the EMS team leaves its base station. The National Fire Protection Association (NFPA), in its codes and standards, NFPA 1710, has set an ECIT of 1 minute or less as the standard.^31^ Indeed, the ECIT will vary for all calls. However, the median ECIT should be at or below the benchmark. An earlier report documented that national ECIT had been on the decline since 2013, from a high of 1 minute 40 seconds to approximately 54 seconds in 2018.^32^ Despite this decline, there are substantial regional and rural‐urban differences in ECIT with a recent study reporting that ECIT in the MidWest urban and rural areas range from 2 to 5 minutes, and 4 to 6 minutes, respectively.^33^ This study found that about two‐thirds of persons exposed to crashes had ECIT of 1 minute or less at all times of the day with the proportion slightly larger during the rush hour compared to the nonrush hour period. However, about half of the persons exposed to crashes in rural areas experienced ECIT longer than a minute compared to a third of those exposed to crashes in urban areas.

To achieve an ECIT of 1 minute or less, there must have been in place an organizational structure that promotes team preparedness while having the appropriate level of resources to address the needs of the population it serves.^34^ The rural‐urban disparity in ECIT may be related to a disparity in resource allocation. Earlier studies have reported on the inequitable distribution of EMS resources and methods of ensuring the equitable distribution of EMS resources using metrics, such as the number of ambulances, the EMS demand, the travel distance, and state‐specific sociodemographic and population health indices.^35^, ^36^, ^37^ Personnel factors that may be associated with increased ECIT may include reduced personnel availability,^38^ the proportion of volunteer services,^33^ and team readiness.^32^ A report had earlier documented how an EMS station reduced its ECIT from an average of 1 minute 42 seconds to an average of 54 seconds through constant discussions around team readiness.^32^ The possibility exists that addressing resource and personnel factors may improve ECIT, which may further reduce the odds of DAS.

The NFPA 1710 had set an ETT of less than 9 minutes as the standard.^31^ However, there are substantial disparities in rural and urban ETT^13^, ^39^, ^40^ with some urban centers achieving ETT of less than 5 minutes, while some rural centers have ETT exceeding 14 minutes.^39^ This study reports that about two‐thirds of persons involved in crashes had ETT less than 9 minutes and this pattern was consistent at all times of the day and during the rush and nonrush hour periods. Additionally, we report that less than 50% of persons involved in crashes in the rural areas had ETT less than 9 minutes compared to over two‐thirds of persons involved in crashes in urban areas.

In this study, the adjusted odds of DAS increased when the ETT fell within the range of 9‐18 minutes compared to less than 9 minutes, and the odds of DAS were heightened during the urban, suburban, and rural rush hour periods. While this study represents the first publicly available study that reports the odds of DAS and ETT, earlier studies have reported the association of prolonged ETT and the odds of fatal crash injury at the individual level^13^ and increased county‐level crash fatality rate ratios.^12^ The rural‐urban disparity in the ETT is associated with the larger landmass rural ambulances cover compared to urban ambulances,^39^ and rural hospital closures.^41^ Optimal citing of EMS centers, especially in regions with prolonged crash response times, may strengthen the EMS nationally and may reduce DAS. Additionally, the use of air ambulance and ambulance drones^40^, ^42^, ^43^ as an adjunct in EMS response, especially in rural and suburban areas, might reduce the ETT and reduce the odds of DAS.

We report that, while ECIT delay between 1 and 5 minutes was associated with increased odds of DAS, ECIT over 5 minutes was associated with reduced odds of DAS. Also, ETT between 9‐ and 18‐minutes window was associated with increased odds of DAS and the odds gradually attenuated to insignificant levels as the ETT increased. The pattern of association between the crash response variables (ECIT and ETT) and DAS remained the same at all times of the day and during the rush and nonrush hour periods. The possible explanation for this may be related to the short window of intervention for resuscitation of crash victims who sustain life‐threatening emergencies. Acute blood loss can reach irreversible levels within 3‐8 minutes, depending on the blood vessels that were damaged. Individuals who experienced delays in ETT and survived life‐threatening crash injuries might have received help from persons around the crash scene.

This study has its limitation. This is a cross‐sectional study, with data pooled over a single study year. Therefore, causal inferences cannot be established. Since the EMS data were pooled across different agencies, the possibility of data entry errors cannot be eliminated. Misclassification of the outcome is highly unlikely since a diagnosis of death is a terminal outcome provided by trained personnel. While the results provide national estimates, NEMSIS data do not include 4 states. However, it is unlikely that these states' results will alter the estimates or disproportionately affect the crash response times or death‐at‐the‐scene. The absence of county‐level identifiers limits the generalization of this result for policy recommendations and practice. Despite these limitations, this study represents one of the few studies that report the proportion of DAS and its association with crash response during the rush and nonrush hour periods.

## CONCLUSION

A small proportion of deaths occur at the crash scene. The proportion of DAS was higher than the proportion of those alive or had non‐DAS deaths when the ECIT was 1 minute or more and the ETT was 9 minutes or more. Increased odds of DAS were significantly associated with EMS crash response times. The odds of DAS were heightened in rural areas and during the rush hour period. Policies and practices aimed at reducing ECIT and ETT may provide early resuscitation at the crash scene and may reduce the occurrence of DAS.

## DISCLOSURES

The authors declare no competing interests.
